# A qualitative investigation into the role of illness perceptions in endometriosis-related quality of life

**DOI:** 10.1177/13591053231183230

**Published:** 2023-06-26

**Authors:** Chloe Moore, Nicola Cogan, Lynn Williams

**Affiliations:** University of Strathclyde, UK

**Keywords:** endometriosis, illness perceptions, qualitative, quality-of-life, wellbeing

## Abstract

Endometriosis is related to adverse quality of life (QoL) and wellbeing outcomes. The way in which endometriosis is perceived by individuals experiencing the condition has not been directly considered, yet illness perceptions (IPs) are predictors of QoL in several chronic conditions. This research aims to gain an understanding of the IPs held by individuals experiencing endometriosis and their impact on QoL. Semi-structured, one-to-one interviews with 30 UK-based participants sought to gain an understanding of participant experiences and perceptions linked to endometriosis. Three themes were constructed through reflexive thematic analysis: a life disrupted; lost sense of self; and complex emotional responses. Largely negative IPs were held by individuals experiencing endometriosis which, along with endometriosis-specific symptoms, fuelled fears for the future and reduced QoL. IP-based interventions may support the QoL of those experiencing endometriosis whilst effective treatment is sought.

## Introduction

Endometriosis is a progressive, incurable condition characterised by the presence of endometrium-like tissue outside the uterus (Chapron et al., 2019). Symptoms include chronic pelvic pain, menorrhagia, dyspareunia, sub-fertility and fatigue, although symptoms vary between those diagnosed with the condition (Kotowska et al., 2021). Endometriosis affects 1 in 10 women and people assigned female at birth globally (World Health Organization, 2021).

Endometriosis has a detrimental impact upon mental health and wellbeing (Wang et al., 2021). Up to 64.4% and 63.5% of individuals diagnosed with the condition experience depression and anxiety respectively (Sepulcri and Do Amaral, 2009), whilst approximately 56% meet the clinical parameters for psychiatric diagnosis (Pope et al., 2015). Endometriosis is further associated with an adverse impact on quality of life (QoL; Kalfas et al., 2022; Roomaney and Kagee, 2018), with 89% of individuals diagnosed with endometriosis describing a negative impact on their ability to lead life as they want (All Party Parliamentary Group, 2020). There is ongoing debate surrounding the mechanisms by which endometriosis leads to reduced QoL.

Pain is perhaps the most prominent driver of endometriosis-related QoL outcomes (Facchin et al., 2015). As pain severity increases, as does the likelihood of stress, anxiety and depression (Facchin et al., 2017), and reduced QoL (Culley et al., 2013). However, there is a lack of effective treatment for endometriosis-related pain and symptomology, so the contribution of other factors to QoL has been examined to ascertain how best to support individuals experiencing endometriosis. Reduced day-to-day functioning (Nnoaham et al., 2011); diagnostic delays (Culley et al., 2013); and coping strategies (González-Echevarría et al., 2019) have been implicated in contributing to reduced QoL in endometriosis. Therefore, rather than one single factor underlying endometriosis-related QoL outcomes, QoL is likely determined by a complex interplay of several physiological, social and psychological factors.

Qualitative research suggests that the way in which individuals perceive their endometriosis, for example, perceived control surrounding the progression and impact of the condition and the anticipated consequences of experiencing endometriosis, is linked to QoL (Jones et al., 2004). A useful framework for examining illness perceptions (IPs) is Leventhal et al.’s (1997) common-sense model of self-regulation (CSM-SR). In this model, IPs are conceptualised as a person’s beliefs and expectations relating to a health condition. The CSM-SR theorises that IPs work with the emotional representation of a health threat to drive behavioural and coping responses (Leventhal et al., 2016). This model situates IPs in five areas: (1) illness identity; (2) expected timeline of the health condition/threat; (3) anticipated consequences; (4) perceived cause of the condition; and (5) perceived control and effectiveness of treatment in regulating or lessening symptoms. Moss-Morris et al. (2002) extended this framework to include a further three IP dimensions: (6) the extent to which an individual understands their condition; (7) the emotional response to the health threat/condition; and (8) concern surrounding the condition. Control was split into two IPs: treatment control and personal control.

IPs contribute to QoL and wellbeing outcomes in several chronic conditions including fibromyalgia (van Wilgen et al., 2008) and irritable bowel disease (Rochelle and Fidler, 2013). Zhang et al. (2016) reported that, for individuals experiencing Crohn’s Disease, IPs directly influenced anxiety, depression, and QoL. Specifically, perceiving negative consequences associated with Crohn’s, and framing the condition as uncontrollable increased the likelihood of depression, anxiety and lowered QoL. Furthermore, interventions targeting IPs have led to modest improvements in treatment outcomes, mental wellbeing, and QoL in several conditions including type 2 diabetes and myocardial infarction (Alyami et al., 2021; Sararoudi et al., 2016), suggesting that such interventions may support the QoL and wellbeing of individuals experiencing endometriosis.

IPs have not yet been directly studied in relation to endometriosis. Previous qualitative literature, however, suggests that beliefs surrounding control and the consequences of endometriosis are related to QoL (Moradi et al., 2014; Young et al., 2015), with more negative perceptions increasing QoL detriments. Therefore, interventions focussed on reframing the IPs of individuals experiencing endometriosis may support QoL in the absence of reliable treatment for endometriosis.

The current study aims to gain an understanding of how endometriosis is perceived and experienced by people diagnosed with the condition, and how these cognitions affect QoL. Findings will be compared against pre-defined IP categories to assess whether the perceptions of people experiencing endometriosis conform to or transcend these categories.

## Method

### Participants

Thirty participants were recruited from a pool of individuals who had completed a survey as part of a broader project investigating factors associated with QoL in endometriosis. These participants were recruited from endometriosis support organisations and social media. A sampling matrix prioritising the recruitment of individuals with a range of ethnic backgrounds, ages, employment status’, household incomes and educational attainment was used to ensure a diverse sample (see Supplemental Material).

Pseudonyms were used to preserve participant anonymity. Participants were aged between 20 and 55 years (*M* = 35.6,) and were eligible to participate if they were diagnosed with endometriosis (e.g. through laparoscopic investigation), were aged 18 years or over, and resided in the UK or Ireland. Details regarding gender were not recorded, however not everyone experiencing endometriosis identifies as female. Therefore, participants are referred to as ‘individuals’ rather than ‘women’ throughout this paper. Twenty participants were white British, whilst the remainder were from various ethnic backgrounds including Indian, African, Pakistani, Asian and mixed backgrounds. Seventeen resided in England, 12 in Scotland and 1 in Wales. Twenty participants were employed, including full-time, part-time and casual employment. Eighteen had co-morbid conditions including gastrointestinal, autoimmune and psychological conditions.

Participants had experienced endometriosis for 4–40 years (M = 14.83 years, SD = 9.18), and were diagnosed for approximately 5 years (SD = 6.97). See Supplemental Material for further demographic information.

### Sample size

A sample size of 30 was deemed appropriate to provide an in-depth, reflective account of participants’ experiences. Data saturation was not considered due to the incompatibility of this concept with Braun and Clarke’s (2021a) reflexive thematic analysis (RTA), which was adopted for this study. Data saturation implies that there is a point at which no new or ‘meaningful’ data can be gleaned from participant accounts, however RTA describes analysis as a fluid and ongoing process with the quality of themes primarily based on the engagement of the researcher with the data rather than the recurrence of similar topics, which is just one element of shaping a theme (Braun and Clarke, 2021a). Codes and themes evolve over the analytical process, and therefore there is no clear endpoint at which codes cease to materialise. Consequently, the sample size for this study was determined pragmatically, based on considerations such as the potential richness of the constructed themes. See Braun and Clarke (2021a) for further discussion on data saturation.

### Data collection

Ethical approval was received from the host institution in April 2021. Subsequently, the sampling matrix was used to identify potential participants who had previously indicated interest in attending an interview. An information sheet was sent to selected individuals via email and those interested provided written consent to be interviewed. Interviews were semi-structured and facilitated online through Zoom (*n* = 25), or telephone (*n* = 5) depending on participant preference. Interviews were audio recorded, lasting 42–90 minutes (M = 62). Audio recordings were deleted following transcription. Participants reiterated their consent verbally before the interview commenced. Following the interview, participants were debriefed and offered a £20 Amazon e-voucher as compensation for their time.

A topic guide consisting of open-ended questions and prompts relating to participants’ perceptions surrounding their condition was developed for the interviews (see Supplemental Material). Questions and prompts related to general perceptions towards endometriosis and specific IPs as outlined by the CSM-SR. The topic guide was piloted with two participants to ensure that questions were relevant and comprehensive. During the interviews, participants described the impact of endometriosis on their lives, before discussing their perceptions of endometriosis. Topics included the consequences associated with endometriosis, the emotional impact, and perceived control over endometriosis.

### Analysis

Transcripts were analysed according to Braun and Clarke’s (2006, 2021b) guidelines for RTA, due to its capacity to reduce large quantities of data into comprehensive, accessible themes that provide a coherent, nuanced account of participant experiences. An inductive approach was adopted to develop themes out-with a theoretical framework, before a deductive approach was taken to compare themes to pre-established IP dimensions. Throughout the analytical process, a reflexive journal was kept by the first researcher to note thoughts, feelings, and assumptions relevant to this process. Initially, the first author read each transcript whilst noting prominent ideas deriving from participant accounts. NVivo was used to organise the data into 124 codes. Potential themes were derived by grouping related codes. Themes were reviewed based on their relevance to the research question before they were defined and named. The final stage of analysis involved discussion amongst all authors regarding the appropriateness of the themes and their definitions in relation to the research question, before three themes were finalised.

## Results

Three themes were constructed through RTA: a life disrupted; lost sense of self; and complex emotional responses. Each theme mapped onto multiple pre-existing IP dimensions (Table 1).

**Table 1.** Identified themes and their relation to IPs.

*Theme*	*Illness perception*
A life disrupted	• **Consequences**: Perceived impact of endometriosis • **Control**: Powerlessness over impact and treatment • **Timeline**: Perception of endometriosis as enduring • **Identity**: Perception of symptoms associated with endometriosis • **Coherence**: Understanding of endometriosis as progressive, incurable • **Emotional representation**: Emotions associated with endometriosis • **Concern**: Concern around impact, progression, future
Lost sense of self	• **Consequences**: Perceived impact of endometriosis • **Control**: Powerlessness over impact and treatment • **Emotional representation**: Emotions associated with endometriosis
Complex emotional responses	• **Timeline**: Perception of endometriosis as enduring • **Identity**: Perception of symptoms associated with endometriosis • **Coherence**: Understanding of endometriosis as progressive, incurable • **Emotional representation**: Emotions associated with endometriosis • **Cause**: Unknown cause, ‘why?’ • **Concern**: Concern around impact, progression, future

### Theme 1: A life disrupted

Participants referred to the multiple and varied life disruptions that were an inevitable consequence of experiencing endometriosis. Many disclosed that endometriosis prevented them from living the life they wanted and felt that their potential in life had not been realised due to the debilitating symptoms they experienced. What constituted participants’ potential varied. Often, potential was defined in terms of career or education goals, although some described their potential in relation to their relationships or fertility. There was a sense of lost time and missed opportunities attributed to endometriosis:

‘*It’s [endometriosis] taken away my youth and it’s taking away from all of the things that I aspire to do and that I could have done and could have achieved*’. [Ash].

This sense of loss was woven through participant accounts, encompassing several life domains including education, work, relationships and day-to-day functioning. No aspect of participants’ lives was untouched by the impact of endometriosis:

‘*It’s an everyday thing that impacts on my actual ability to just function in life*’. [Morgan].

The language used by participants implied a sense of powerlessness attributed to endometriosis, which manifested itself in participants’ life trajectories. Several perceived endometriosis as ‘controlling’ [Alina] their lives, and viewed their life trajectories as dictated by the progression of the condition:

‘*I’m not in control of my life, this illness is*’. [Billie].

Perceived disruption to life trajectories often triggered negative emotions. Participants described sadness and frustration related to their circumstances, with some experiencing anxiety and depression attributed to endometriosis-related life disruption(s). Adverse QoL outcomes were experienced by several participants, largely facilitated by day-to-day functioning detriments triggered by endometriosis:

‘*It’s actually not the pain that’s the worst part of the disease, it’s the impact that it has on my life and how it prevents me from doing things that has affected my mental health more than anything*’. [Emily].

This impact on wellbeing was associated with several life domains including work and relationships. Negative wellbeing outcomes were particularly pertinent for participants experiencing uncertainty around their fertility. Importantly, participants differed widely in their thoughts, feelings, and experiences surrounding fertility and parenthood, with several childfree by choice, some actively trying to conceive and others who had successfully conceived. Nonetheless, several participants described feelings of helplessness, powerlessness, and anxiety related to their fertility, particularly when conceiving was difficult or unsuccessful:

‘*I was in tears yesterday because it’s getting faced with that reality of- I would love a family but I might not be able to have one*’. [Mira].

Beyond fertility, there was a general sense of fear and apprehension surrounding the future woven throughout participant accounts. Fear was often fuelled by historic disruptions to participants’ life trajectories (e.g. in work, relationships), and the incurability of endometriosis, leading many to speculate that their symptoms would last forever. Participants demonstrated awareness of the progressive nature of their condition, often driving fears that their symptoms might worsen and cause further disruption to their lives:

‘*It can grow as it likes, it has no cause that’s known, there’s no treatment plan, the pain is excruciating and it will never go away. And I think that to me felt like, this thing is going to colonise my life*’. [Polly].

However, participant responses were complex and diverse, and correspondingly some did not share the apprehension towards the future as described above. Several described ‘taking one day at a time’ [Jackie] and focussing on the present to prevent fears surrounding the future from taking hold:

‘*I don’t look too far ahead because this time 6 months ago I was in a completely different place so, you know, I just take every day as it comes and make the most of it*’. [Violet].

Similarly, many had found ways to live with the life disruptions associated with endometriosis, for example by seeking employment with flexible working patterns, or keeping diaries of their symptoms and triggers to pre-empt endometriosis flare-ups and prepare for impending symptoms. Participants utilised several coping strategies to minimise the adverse mental health impact associated with endometriosis-related life disruption:

‘*I now have a coping style. I think for a long time I was just not coping, and not even knowing that I wasn’t coping*’. [Casey].

Clear through each participant’s account was their resolve to regain the control thought lost through endometriosis:

‘*I’m going to be able to live with this, it’s not going to take my life’*. [Sarah].

### Theme 2: Lost sense of self

Participants generally considered their identity as moulded by endometriosis. Several described their sense of self as ‘lost’ [Violet], often stating that they felt like a ‘different person’ [Robin] owing to the impact of endometriosis on their lives:

‘*I don’t think I’ll ever be the person I was, I think this [endometriosis] has changed me forever’*. [Becky].

Some described the heavy emotional burden associated with experiencing a progressive, often debilitating condition. There was a sense that endometriosis slowly eroded the sense of self, that the longer this emotional burden was carried, the more significant the impact on the self-concept:

‘*. . .having to put up with years of pain and sort of like losing yourself to the disease, you feel like you’re not yourself anymore. You’re someone different. You become the illness in a way*’. [Billie].

This sense of the self as lost to endometriosis was often interlinked with feelings of vulnerability elicited by the symptoms associated with endometriosis. For many, pain and fatigue progressed with the condition, provoking a shift in self-perception. Some defined themselves as increasingly ‘sick’ [Indra], ‘unwell’ [Alex], and there was a sense amongst some that endometriosis had eroded previously salient aspects of their identity:

‘*I did change and I became a victim. Um, and that wasn’t me before, you know, I was always independent, stood on my own two feet and didn’t rely on anybody and with, with the pain and everything else I became a different person*’. [Robin].

Perceptions of the self as increasingly unwell, along with functioning detriments engendered by endometriosis symptoms, prompted shifts in specific aspects of the identity. Femininity and sexual identity were particularly impacted by endometriosis and were intrinsically linked, in that specific symptoms (e.g. dyspareunia, bloating) eroded participant’s sexual drive which dismantled perceptions of femininity. This sense of diminished femininity then impacted sexual desire:

*‘I just physically can’t [have sex]. I don’t feel feminine, I don’t feel sexy because I’m in pain*’ [Nathalia].

Participants addressed the pain associated with sex in several ways, including ‘putting on a performance for male partners’ [Emily] and avoiding sexual activity. Several described feelings of guilt and shame surrounding sex:

*‘We haven’t done anything for like a year, maybe more now. And I feel so guilty. I deal with that guilt every day*’ [Morgan].

Correspondingly, several participants perceived themselves as ‘less of a woman’ [Billie; Emily] due to the impact of endometriosis-related symptoms on their sexual functioning and feminine identity. However, several had taken positive steps to regain their sexual identity, including sexual experimentation, and moving away from traditional definitions of sex:

*‘’We haven’t had penetration in our sex life and it hasn’t impacted our intimacy [. . .]. Em, we can both experience pleasure that doesn’t involve me being in agony and crying because that isn’t fun for anyone. Re-framing that definition* [of sex] *has helped me to feel better about myself as a woman’.* [Emily].

As demonstrated by this extract, restructuring pervasive and negative perceptions around the destructive nature of endometriosis can lead to improved self-esteem and wellbeing.

Importantly, not everyone who experiences endometriosis identifies as feminine or female. One non-binary participant described the impact of living with endometriosis on their own self-perception. Living with symptoms such as menstrual bleeding and chronic pelvic pain activated a sense of gender dysphoria in them, leaving them ‘confused’, ‘isolated’ and with a fragmented sense of their own gender identity:

*‘[diagnosis] didn’t help with the old gender identity because they’re very much, this is a woman’s disease, this is a woman’s illness. This is a thing that happens to women. And I’m just over here like, oh no’.* [Morgan].

Shifts in the self-concept were not only linked to endometriosis symptomology, but the broader treatment of endometriosis in societal and medical settings. There was a sense that symptoms were minimised and dismissed by others, particularly healthcare professionals, leading to feelings of invalidation and a sense of being silenced by the institutions participants had placed their trust in:

*‘*. . .*just trying to get the doctors to accept that I’m saying no, this isn’t right, it [sigh]- they’ve left me feeling on more than one occasion completely invalidated about it*’. [Mira].

Several participants described the internalisation of these minimised symptoms, leading to the belief that what they were experiencing was characteristic of menstruation and the result of a ‘low pain threshold’ [Iona; Robin]. Participants frequently compared themselves to other menstruating individuals who appeared to have no menstruation-related problems, leading often to self-chastisement and frustration:

*‘I remember looking around me and wondering, you know, how come other girls seem to have no problem with going to school on their periods? [. . .] How come I can’t seem to function like a normal human being?’* [Emily].

Through the internalisation of minimised symptoms, participants frequently voiced a sense that they did not know themselves. After repeated instances of their concerns being minimised and/or disregarded, many questioned their intuition and even their own ‘sanity’ [Emily]. For several participants, this led to confusion and anxiety:

*‘I was just thinking, I’m imagining these pains, there is no pain here, I’m just imagining it’.* [Charlie].

Subsequently, there was often a sense of an internal struggle to control the self-concept, in which internalised notions that symptoms were not real or were a ’normal’ part of menstruating were pitted against a sense that symptoms were real and valid:

*‘You feel like something’s there but you keep getting told that nothing’s there and then it’s this anxiety of, I’m imagining things. I don’t know what’s real and what’s not anymore*’. [Indra].

Despite the wide-reaching impact of endometriosis on the self-concept, there was a determination not to allow endometriosis to completely seize the identity:

*‘it doesn’t define me, this is just something that I deal with and I cope with*’. [Sarah].

Additionally, some reflected on the positive ways in which endometriosis had shaped their identity, specifically highlighting patience, resilience and strength:

*‘it’s only recently that I’ve looked back on everything and thought you know what, I am strong, [. . .] I’ll push for things and I’m brave and I’ll talk about things and I’m not shy about it and, um, you know, I think that’s sort of changed my identity*’. [Alina].

### Theme 3: Complex emotional responses

Participants’ endometriosis-related experiences prompted several emotions. Emotions varied widely but can be separated into two categories: (i) endometriosis as an emotional burden; (ii) endometriosis as a facilitator of emotional strength. Participants generally described emotions in each of these categories, highlighting the complexity of their feelings surrounding endometriosis.

#### Endometriosis as an emotional burden

Frustration was the most prominent emotion described in relation to participants’ experiences of endometriosis. This often stemmed from knowledge of the incurable and progressive nature of endometriosis, and the lack of effective treatment. The unknown cause of endometriosis also elicited feelings of frustration:

‘*How are there people that don’t end up suffering? Obviously you wouldn’t wish it on anyone but it’s just that understanding of why certain people get it and why other people don’t and it makes you feel frustrated*’ [Jenny].

Widespread misunderstanding and the minimisation of participant experiences in medical settings was also a common source of frustration:

‘*It’s been frustrating that no-one would take me seriously, frustrating that lead times on appointments were too long, frustrating that, you know, it’s something we’ve known about for hundreds of years and yet we still don’t know anything about it’.* [Becky].

Frustration preceded feelings of anger for many participants. Anger was often intertwined with the anticipated negative impact of endometriosis on the life trajectory and identity, and linked with feelings of powerlessness:

‘*I am raging inside that I’ve got to be kind of forced into a position of being weak and not being able to do what I want to do*’. [Reece].

Feelings of sadness were also described by several participants. As above, sadness tended to revolve around a sense of powerlessness over endometriosis. Participants often voiced a sense of being ‘attacked’ [Ava] by their own body, leading to hopelessness:

‘*Sometimes I’m like I can’t believe my body is betraying me, it’s like really just rubbish, why does, you know, so that’s quite, I would say, a little bit upsetting* [crying]’. [Evelyn].

Guilt was another prominent emotion throughout participant accounts. Guilt tended to surround the impact of endometriosis on relationships, for example, being unable to engage in sexual activity with partners or rejecting social invitations due to endometriosis-related symptoms. Participants with daughters often shared a sense of guilt and dread at the prospect of their children inheriting the condition:

‘*What kills me is I’ve just had a baby, and when I found out it was a girl it was definitely in my head that this is something that I’m now going to pass on to her and she’s now going to have to live with this and that made me upset*’. [Sarah].

Furthermore, participants described feelings of loneliness, often prompted by a sense that their experiences were misunderstood within societal and medical settings due to a lack of understanding and education surrounding endometriosis:

‘*I have went for the better part of about 13 years going no-one else experiences what I experience. Having no one else that understands it is very, very isolating*’. [Mira].

For some, the emotional pain they experienced due to endometriosis progressed into longer-term mental health concerns. Many described long periods of low mood and depressive symptoms:

‘*It [endometriosis] affected my mental health big time. I woke up in the morning and just felt like there was this black cloud above my head and I didn’t want to get up, I just wanted to hide away*’. [Billie].

#### Emotional strength stemming from endometriosis

Contrarily, some participants described finding emotional strength through their experiences of endometriosis. This strength was often forged through establishing coping mechanisms to minimise the mental health impact of endometriosis:

‘*I think it’s had to make me a stronger person because I’ve just had to deal with it, it’s just something that, that’s part of my life*’. [Sarah].

The notion of ‘dealing with’ endometriosis implies a sense of control over the condition, indicating that emotional strength may be derived from challenging the feelings of powerlessness that are so often linked to endometriosis.

Participants often derived feelings of empowerment and strength through using their experiences to support and advocate for others. For many, this gave value and purpose to their experiences:

‘*I’ve done a lot of work helping other people which has gave me a purpose and something good that’s come out of it where I’ve been able to help other people whether it’s to give advice and support or just to listen and tell them ‘I understand what you’re going through’*’. [Emily].

‘*It’s almost like having a little piece of wisdom that you get from unfortunate circumstances”*. [Alina].

One participant described endometriosis as giving them a sense of ‘pride’ [Casey]. Benefit finding was common amongst participants, indicating a determination to mitigate against the negative emotional impact associated with the condition:

‘*[Endometriosis] has taught me a lot of patience. I like myself more now. So, that’s a good thing*’. [Jessie].

## Discussion

This study is the first to qualitatively explore endometriosis-related IPs and their relation to QoL amongst individuals experiencing endometriosis. An inductive and deductive approach to analysis allowed for IPs to be considered both organically and within a theoretical framework.

Broadly, the findings reflect previous research suggesting that endometriosis has a detrimental impact on the QoL and wellbeing of those experiencing the condition (Wang et al., 2021). There were, however, disparities within participant accounts regarding the extent to which endometriosis impacted aspects of QoL, with some recounting a pervasive, debilitating effect on their lives, and others describing a more manageable, fluctuating impact. This nuance in participant experience is likely associated with disparities in endometriosis symptomology. Although the findings of this study conform to the notion that endometriosis symptomology is inherently linked to QoL, they add to the existing literature by highlighting additional mechanisms by which endometriosis may impact QoL and wellbeing, specifically by moulding IPs which, within this participant group, were linked to dimensions of QoL such as the life trajectory, identity, and wellbeing. IPs were shaped directly by endometriosis symptomology and indirectly through functioning detriments.

Each inductively identified theme mapped on to multiple pre-defined IPs (Table 1). Research has already demonstrated the pervasive, negative impact of endometriosis on life domains such as relationships, careers, and sex and fertility (Halici et al., 2023; Missmer et al., 2021). Theme 1 encapsulates these effects, demonstrating the wide-ranging negative anticipated and actual consequences of endometriosis on participants’ life trajectories. Participants highlighted specific symptoms such as pain and fatigue as the cause of disruption to their expected life trajectories, indicating a lack of control over endometriosis and the subsequent impact on their lives. This is perhaps unsurprising, given the incurability of endometriosis and research demonstrating that treatment is often ineffective (Nirgianakis et al., 2020). Indeed, participants demonstrated a strong awareness and knowledge of their condition, including the incurable nature of endometriosis and the potential progression of symptomology, and this was instrumental in cultivating feelings of powerlessness. Furthermore, participants believed that endometriosis symptoms would persist throughout their lifespan. Perceptions of the enduring timeline of endometriosis were linked to fears associated with the consequences of endometriosis on life outcomes and perceptions of control over the condition. Research suggests that endometriosis symptoms persist even after menopause (Secosan et al., 2020), potentially fuelling the fears for the future voiced by participants. The perceived consequences of endometriosis on the life trajectory prompted a strong emotional response from many participants, who described detrimental wellbeing effects stemming from the disruption and anticipated disruption to their lives, including anxiety and sadness. However, importantly, some participants described re-framing their perceptions around the consequences of endometriosis, leading to improvements in their self-esteem. It is important that future research investigates this potential link further to establish whether interventions to re-frame IPs may be beneficial for individuals experiencing endometriosis.

Corresponding with research suggesting a link between endometriosis and the identity (Cole et al., 2021), theme 2 highlights a fragmented and lost sense of self attributable to endometriosis. Although identity is a pre-defined IP, ‘identity’ within this theme transcends the CSM-SR’s definition, in which it is centred around perceptions of the symptoms associated with the condition rather than the sense of self. In this study, identity refers to the broader self-concept and theme 2 explores participants’ perceptions of how this is moulded by endometriosis. This is interlinked with theme 1, as many of the perceived changes to identity stemmed from the impact of endometriosis on life domains such as work and relationships. This corresponds with research demonstrating that the sense of self is intrinsically linked to social aspects including career choice (Fryers, 2006), relationships (Andersen and Chen, 2002) and sex (Hensel et al., 2011). Correspondingly, the self-concept is linked to QoL in other chronic conditions (Octari et al., 2020), suggesting that IPs may indirectly impact endometriosis-related QoL by shaping the identity. Additional research is required to examine this potential link further.

Within this study, participants used terms such as ‘lost’ to describe their identity, implying a sense of powerlessness surrounding their sense of self. However, using the term ‘lost’ rather than, for example, ‘broken’ or ‘gone’ suggests a sense that the self-concept may be recovered, as found among people experiencing other chronic conditions (Cogan et al., 2019; Golub et al., 2014). This implies an underlying hope that control of the identity might be regained from endometriosis. This corresponds with the dichotomy observed within some participant accounts, in which the self-concept was described as driven by endometriosis but, simultaneously, there was a determination to prevent endometriosis from taking over the identity.

Examining participant’s perceptions of their identity through an IP lens revealed shared experiences amongst participants such as the internalised trivialisation of endometriosis-related symptomology, which corresponds to broader social themes including the treatment of women’s health conditions in medical and societal environments. There is vast sociological discourse on the treatment of women’s health conditions that corresponds with participant accounts of the minimisation of their symptoms at both societal and medical levels (Alexander et al., 2020). Within this study, participants often questioned their knowledge and expertise in their bodies, with some doubting their experiences and even their ‘sanity’. Thus, many appeared to experience a sense of externalised self-perception (Jack and Dill, 1992) in which they viewed themselves through the lens of others. This is described as an act of self-silencing, which may be activated when individuals experience a strong fear of rejection through voicing their own thoughts and feelings (Jack and Dill, 1992). Self-silencing is associated with an underlying fear that internal thoughts and feelings are inaccurate, particularly when they contradict societal norms (Maji and Dixit, 2019). As stigma surrounds menstruation, self-silencing is particularly pertinent to endometriosis (Cole et al., 2021) and is evident within participant accounts throughout this paper.

Similarly, sex was often related to the feminine identity and notions of ‘womanhood’, which relates to broader sociological discourse surrounding femininity and sexual pleasure (Carter et al., 2019). Importantly, participants in heterosexual relationships often equated sex to penetration, but in moving away from traditional definitions of sex to find alternate ways to enjoy sexual activity, some participants experienced increased confidence and intimacy with their partners. This suggests that interventions focussed on reframing traditional definitions of sex may be beneficial for individuals experiencing endometriosis with dyspareunia. Future research should further investigate subjective definitions and experiences of femininity and the intersection between perceptions of femininity and sexual drive in endometriosis.

A strong emotional response to endometriosis was woven throughout participant accounts, and this is described in theme 3. Feelings of anger and frustration correspond to previous qualitative research where they are often intertwined with endometriosis-specific factors such as treatment effectiveness and diagnostic delay (Jones et al., 2004). Within this study, the emotional response was interlinked with perceptions of control, coherence, consequences and the anticipated longevity of endometriosis symptoms. Negative emotional responses were prominent throughout participant accounts, corresponding with previous literature suggesting that frustration, fear, and sadness are common amongst people experiencing endometriosis (Young et al., 2015). However, perhaps surprisingly, some participants described positive emotions associated with endometriosis, emphasising resilience, pride and strength cultivated by their experiences. This was often linked with a sense of hope for the future and participants finding value in their experiences by supporting and advocating for others. In this participant sample, benefit finding was commonly used to lessen the emotional impact of endometriosis. The impact of benefit finding on emotions and wellbeing has not been researched in endometriosis and therefore constitutes an important area for future study.

IPs in this participant sample could be matched to each of the pre-defined IPs as described in the CSM-SR (Table 1). Most prominent in this sample were perceptions of control and consequences, which were clearly linked to participants’ life trajectories, self-concept, and emotional response. Less clear however was the role of the illness identity (i.e. the symptoms associated with endometriosis by participants) or the perceived cause of endometriosis. As there is no known cause for endometriosis, perceptions around causation may not be particularly strong within this population which may be reflected in the acquired results. However, research suggests that many individuals experiencing endometriosis do hold subjective views of the cause of their condition (Münch et al., 2022), and in the current research the absence of a known cause often prompted a negative emotional response. Therefore, future research could endeavour to establish whether there is a link between perceptions of endometriosis cause and QoL and/or wellbeing.

Considering the findings of this research, namely that the experiences of participants are linked to IPs, and that shifts in some IPs appear to prompt positive QoL and psychological outcomes, it is possible that IP-based interventions may partially mitigate the detrimental impact of endometriosis on QoL outcomes. This is not to say that psychological intervention can replace effective, symptom-targeted treatment, but that it may support the wellbeing of individuals with endometriosis whilst reliable treatment is sought. Due to the dearth of research on this topic, future research could assess IPs with a large sample of individuals using pre-established measures of IPs such as the revised illness perception questionnaire (Moss-Morris et al., 2002) to investigate further the appropriateness of studying endometriosis within a CSM-SR framework before corresponding interventions are trialled.

### Strengths and limitations

To our knowledge, this is the first study to qualitatively consider the IPs of individuals experiencing endometriosis. This paper extends current knowledge on the mechanisms underlying adverse QoL outcomes in endometriosis by suggesting that IPs may contribute to QoL alongside stronger predictors of wellbeing such as pain. By exploring participant experiences through an RTA framework, this paper offers a rich narrative of the experiences of individuals living with endometriosis, complementing the literature highlighting the pervasive and enduring impact of endometriosis.

However, this study must be viewed in light of its limitations as well as strengths. Firstly, participants were recruited through social media and support groups, indicating that many had sought support for their condition. People involved in support groups may hold views unreflective of the wider population in two distinct ways: (i) they may have worsened symptomology and more negative experiences leading them to seek support; (ii) they may have a more positive outlook surrounding their diagnosis due to increased support. Furthermore, several participants (60%) experienced co-morbid conditions, so functioning and QoL detriments may be attributable to living with multiple medical conditions rather than endometriosis alone. However, the potential impact of this may be mitigated as interviews focussed solely on endometriosis rather than general health.

Additionally, the interview topic guide was underpinned by the CSM-SR and many questions related to pre-existing IPs. Therefore, although an inductive approach was taken in constructing themes, the information yielded from the interviews may have been heavily slanted towards the CSM-SR’s depiction of IPs. Therefore, important IPs held by participants but existing out-with this theoretical framework may have been missed and the role of pre-established IPs over-emphasised. However, in investigating IPs within a pre-established framework, this research lays the groundwork for future investigation into the role of IPs in endometriosis-related outcomes by suggesting that these cognitions likely contribute to QoL and wellbeing outcomes.

Furthermore, participants resided in the UK. Due to cultural differences in attitudes towards menstruation, the results may therefore not reflect the experiences of individuals residing elsewhere. Therefore, future research could consider whether the IPs of individuals experiencing endometriosis are universal or differ between cultures.

Finally, participants were not asked to disclose their gender. One participant identified as non-binary and discussed the isolation and loneliness they felt due to experiencing endometriosis as a non-cisgender individual. Future qualitative research should endeavour to increase understanding around the barriers and issues faced by non-cisgender individuals in accessing endometriosis-related support.

## Conclusion

This study highlights the complex and dynamic nature of the IPs held by individuals experiencing endometriosis. Endometriosis-specific symptoms such as pain were the main driver of QoL detriments, and these symptoms and their associated impact cultivated and moulded endometriosis-related IPs. Whilst effective treatment is sought for endometriosis-related symptoms, research should continue to investigate the factors that may mitigate the detrimental impact of endometriosis on QoL and wellbeing. These findings offer clear indications that interventions based on endometriosis-related IPs may support the QoL of individuals experiencing endometriosis, and suggests that future research explore the link between IPs and QoL in endometriosis further.

## Supplemental Material

sj-docx-1-hpq-10.1177_13591053231183230 – Supplemental material for A qualitative investigation into the role of illness perceptions in endometriosis-related quality of life Click here for additional data file. Supplemental material, sj-docx-1-hpq-10.1177_13591053231183230 for A qualitative investigation into the role of illness perceptions in endometriosis-related quality of life by Chloe Moore, Nicola Cogan and Lynn Williams in Journal of Health Psychology

sj-docx-2-hpq-10.1177_13591053231183230 – Supplemental material for A qualitative investigation into the role of illness perceptions in endometriosis-related quality of life Click here for additional data file. Supplemental material, sj-docx-2-hpq-10.1177_13591053231183230 for A qualitative investigation into the role of illness perceptions in endometriosis-related quality of life by Chloe Moore, Nicola Cogan and Lynn Williams in Journal of Health Psychology

sj-docx-3-hpq-10.1177_13591053231183230 – Supplemental material for A qualitative investigation into the role of illness perceptions in endometriosis-related quality of life Click here for additional data file. Supplemental material, sj-docx-3-hpq-10.1177_13591053231183230 for A qualitative investigation into the role of illness perceptions in endometriosis-related quality of life by Chloe Moore, Nicola Cogan and Lynn Williams in Journal of Health Psychology
