# Supplementary material for: A qualitative investigation into the role of illness perceptions in endometriosis-related quality of life
Source: J Health Psychol. 2023 Jun 26;28(12):1157–71. doi: 10.1177/13591053231183230 (PMC10571435; doi:10.1177/13591053231183230)
Supplement: sj-docx-1-hpq-10.1177_13591053231183230 – Supplemental material for A qualitative investigation into the role of illness perceptions in endometriosis-related quality of life [file sj-docx-1-hpq-10.1177_13591053231183230.docx]

**Table 1**

| *Participant demographics* | |  |
| --- | --- | --- |
|  | N | % |
| **Ethnicity** |  |  |
| White British | 20 | 66.7% |
| Indian | 2 | 6.7% |
| African | 2 | 6.7% |
| Another Mixed Background | 2 | 6.7% |
| Pakistani | 1 | 3.3% |
| Asian and White | 1 | 3.3% |
| Another Ethnic Background | 1 | 3.3% |
| Another White Background | 1 | 3.3% |
|  |  |  |
| **Relationship Status** |  |  |
| Married | 10 | 33.3% |
| Cohabiting with partner | 10 | 33.3% |
| Single | 9 | 30% |
| Widowed | 1 | 3.3% |
|  |  |  |
| **Educational attainment** |  |  |
| Undergraduate / Bachelors degree | 10 | 33.3% |
| Postgraduate degree | 6 | 20% |
| Secondary education to GSCE/O-levels/National 5 or equivalent | 4 | 13.3% |
| Secondary education to Highers/A-level or equivalent | 3 | 10% |
| Diploma of Higher Education/Foundation Degree/Higher National Diploma/NVQ level 5/level 5 diploma or equivalent | 3 | 105 |
| Left school with no qualifications | 2 | 6.7% |
| Completed secondary school to National 3/4 or standard grade | 1 | 3.3% |
| Prefer not to say | 1 | 3.3% |
|  |  |  |
| **Employment status** |  |  |
| Employed full-time | 11 | 36.7% |
| Disabled or unable to work | 6 | 20% |
| Employed part-time | 5 | 16.7% |
| Self-employed | 3 | 10% |
| Unemployed, looking for work | 2 | 6.7% |
| Employed on a zero hours or casual contract | 1 | 3.3% |
| Full-time student | 1 | 3.3% |
| Part time student | 1 | 3.3% |
|  |  |  |
| **Country of residence** |  |  |
| England | 17 | 56.7% |
| Scotland | 12 | 40% |
| Wales | 1 | 3.3% |

**Table 2**

| *Participant endometriosis information* | |  |
| --- | --- | --- |
|  | N | % |
| **Treatment** |  |  |
| NHS | 16 | 53.3% |
| Some private, some NHS | 13 | 43.3% |
| Completely private | 1 | 3.3% |
|  |  |  |
| **Co-morbid condition** |  |  |
| Yes | 18 | 60% |
| No | 12 | 40% |
|  |  |  |
| **Surgery** |  |  |
| Had surgery | 24 | 80% |
| Not had surgery | 6 | 20% |
|  |  |  |
| **Number of surgeries** |  |  |
| 1 | 10 | 33.3% |
| 2 | 8 | 26.7% |
| 3 | 3 | 10% |
| 4 | 2 | 6.7% |
| 5 | 1 | 3.3% |
|  |  |  |
| **Trying for baby** |  |  |
| Yes | 12 | 40% |
| No | 17 | 56.7% |
| Prefer not to say | 1 | 3.3% |
